# No evidence of genetic causation between iron and infertility: a Mendelian randomization study

**DOI:** 10.3389/fnut.2024.1390618

**Published:** 2024-07-22

**Authors:** Liangliang Guo, Shengnan Yin, Hongkui Wei, Jian Peng

**Affiliations:** ^1^Department of Animal Nutrition and Feed Science, College of Animal Science and Technology, Huazhong Agricultural University, Wuhan, China; ^2^The Cooperative Innovation Center for Sustainable Pig Production, Wuhan, China; ^3^Frontiers Science Center for Animal Breeding and Sustainable Production, Wuhan, China

**Keywords:** iron, infertility, men, women, Mendelian randomization

## Abstract

**Background:**

Observational studies have explored the impact of iron homeostasis on infertility; however, establishing definitive causal relationships remains challenging. This study utilized a two-sample Mendelian randomization approach to investigate the potential causal relationship between iron status and infertility.

**Materials and methods:**

Four indicators of iron status-serum iron, ferritin, transferrin saturation, and total iron binding capacity, were considered as exposure factors. Infertility was the outcome variable for both men and women. Robust causality was assessed using the primary inverse-variance-weighted method, complemented by three supplementary Mendelian randomization approaches. Sensitivity analyses were performed to enhance the precision and reliability of the results.

**Results:**

No statistically significant associations were identified between the four indicators of iron status and infertility. These results remained consistent across multiple Mendelian randomization methodologies.

**Conclusion:**

In conclusion, there is no evidence of a genetic causal relationship between iron status and infertility. Nevertheless, this does not preclude the possibility of a connection between iron status and infertility at different mechanistic levels.

## Introduction

Infertility, defined as the inability to conceive after 12 months of regular, unprotected sexual intercourse, is a pervasive global health concern (1). It is estimated that approximately 15% of the global population is affected by this condition, prompting extensive discourse and research efforts (2). Infertility exerts a dual impact, profoundly affecting both individuals and their families, while also imposing a significant economic strain on society. This condition can stem from various factors, including male or female reproductive issues, as well as associations with diseases (3), lifestyle choices (4), nutritional deficiencies (5), and other yet-to-be-discovered elements (6). Therefore, it is essential to pinpoint the underlying factors causing infertility to develop better prevention strategies.

Iron, though a trace element, plays a pivotal role in essential metabolic processes crucial for human health, including oxygen transport, DNA synthesis, and ATP production (7). Indicators of iron status, including serum iron, ferritin, transferrin saturation (TSAT), and total iron-binding capacity (TIBC), are crucial for reflecting the metabolism and utilization of iron in the body (8). Serum iron directly reflects the level of free iron in the body but fluctuates physiologically (9). Ferritin is the most reliable and sensitive indicator of iron deficiency in the body (10). TIBC, the maximum capacity of serum transferrin to bind iron, indirectly reflects serum transferrin levels (11). TSAT, determined by the ratio of serum iron to TIBC, provides insight into the circulating iron available (12). Research suggests that women diagnosed with infertility typically have iron deficiency (13). A recent study on recurrent pregnancy loss indicated that women prone to miscarriage have lower serum iron levels and are more likely to have low iron status (14). In men, iron is essential for spermatogenesis (15), and low serum iron levels can impair fertility and lead to poor semen quality (16). However, elevated iron levels can induce oxidative stress, which may significantly contribute to idiopathic infertility (17, 18). Thus, maintaining iron homeostasis is crucial for reproductive health. However, the causal link between iron status and infertility remains unclear.

The association between risk factors and outcomes in observational studies is susceptible to the influence of age, environment, mental state, lifestyle, and other confounding factors (19). Utilizing single nucleotide polymorphisms (SNPs) as instrumental variables (IVs), Mendelian randomization (MR) analysis, is widely used to investigate causal relationships, mitigating bias caused by confounding factors through randomly segregated alleles (20, 21). Numerous studies have demonstrated the utility of MR methods in investigating causal relationships between risk factors and outcomes, such as the association between iron status and osteoarthritis (22) and the causal link between gut microbiota and infertility risk (23). However, there is a scarcity of research specifically investigating the connection between iron status and infertility using MR analysis. To elucidate this relationship, we conducted a two-sample MR analysis using genome-wide association study summary statistics to investigate the causal effects of iron status on infertility.

## Materials and methods

### Study design

**Figure 1:**
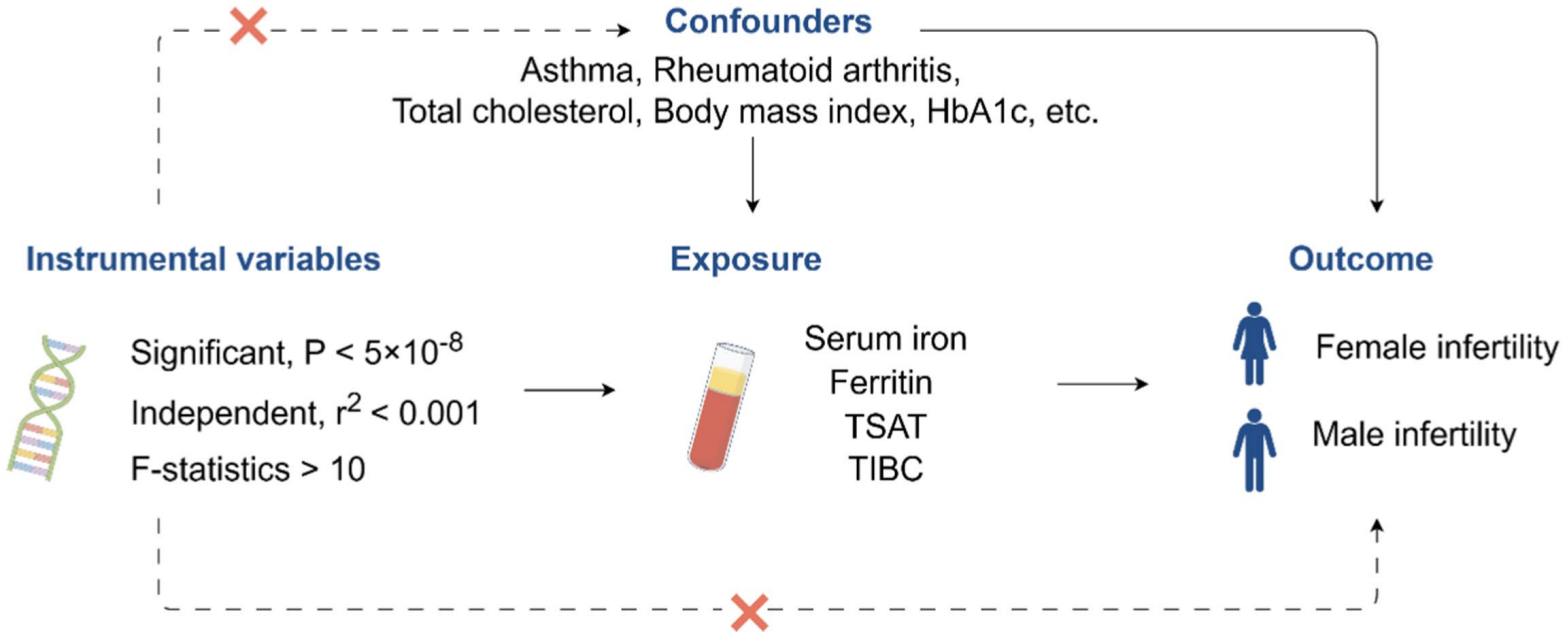
Workflow for this study. TSAT, transferrin saturation; TIBC, total iron-binding capacity; HbA1c, glycated hemoglobin. The image was drawn by Figdraw (https://www.figdraw.com).

We conducted a two-sample MR analysis to assess the causal relationship between iron status and infertility. The workflow of this study is illustrated in Figure 1.

### Data sources

For this study, all datasets were sourced from publicly available databases. Genetic variants data related to iron status, including serum iron, ferritin, TSAT, and TIBC, were obtained from a meta-analysis of a genome-wide association study (8). This meta-analysis amalgamated data from three cohorts in Iceland, the United Kingdom, and Denmark, with information on serum iron (*n* = 163,511), ferritin (*n* = 246,139), TSAT (*n* = 131,471), and TIBC (*n* = 135,430) detailed in Supplementary Table S1. Genetic variants linked to both male and female infertility were extracted from the FinnGen Consortium version R9.^1^ The summary statistics for female infertility comprised 13,142 cases and 107,564 controls, while those for male infertility included 1,271 cases and 119,297 controls, as outlined in Supplementary Table S1 (24). The diagnosis of female infertility in FinnGen is defined by the International Classification of Diseases 10th Revision (ICD-10) code N97, which includes infertility caused by issues with the ovaries, fallopian tubes, and uterus. The diagnosis of male infertility is defined by the ICD-10 code N46, which includes azoospermia and oligospermia.

### Instrumental variable selection

To ensure the reliability of the MR analysis, we strictly adhered to the following principles when selecting instrumental variables: (1) SNPs were chosen as IVs based on a threshold of *p* < 5 × 10^−8^ for the four iron indicators. (2) The linkage disequilibrium between IVs was removed (clumping distance = 10,000 kb and clumping r^2^ < 0.001) (25, 26). (3) The F-statistics of the selected IVs surpassed the conventional threshold of 10. After preliminary filtering, 16, 51, 19, and 26 SNPs corresponding to serum iron, ferritin, TSAT, and TIBC were selected. We then used the PhenoScanner website^2^ to retrieve SNPs with potential associations with both exposure and outcome. Asthma (3), rheumatoid arthritis (27), total cholesterol (28, 29), body mass index (30, 31), glycated hemoglobin (32, 33), and vitamin B12 levels (34, 35) have been reported to be associated with infertility. Therefore, we removed these confounders to exclude the possibility of genetic pleiotropy. Eventually, 15, 43, 17, and 20 SNPs corresponding to serum iron, ferritin, TSAT, and TIBC were used in the subsequent analysis (Supplementary Table S2). Additionally, we specifically list the above SNPs in Supplementary Table S3.

### Statistical analysis

Four methods, including inverse variance weighted (IVW), MR-Egger, weighted median, and weighted mode, were utilized to investigate the causal link between iron status and infertility. IVW was utilized as the primary analytical approach due to its ability to yield unbiased results in the absence of pleiotropy (36, 37). Furthermore, the inclusion of three supplementary methods aimed to ensure more robust findings across diverse scenarios. Specific sensitivity analyses, encompassing heterogeneity tests, pleiotropy tests, and MR-Pleiotropy Residual Sum and Outlier (MR-PRESSO) tests, were conducted to evaluate and address potential issues related to heterogeneity and pleiotropy (38). MR analysis was performed using the “TwoSampleMR” package (39) in R software version 4.1.3. Radial plots of the MR estimates were prepared using the “RadialMR” package (40).

## Results

### Mendelian randomization analysis

**Figure 2:**
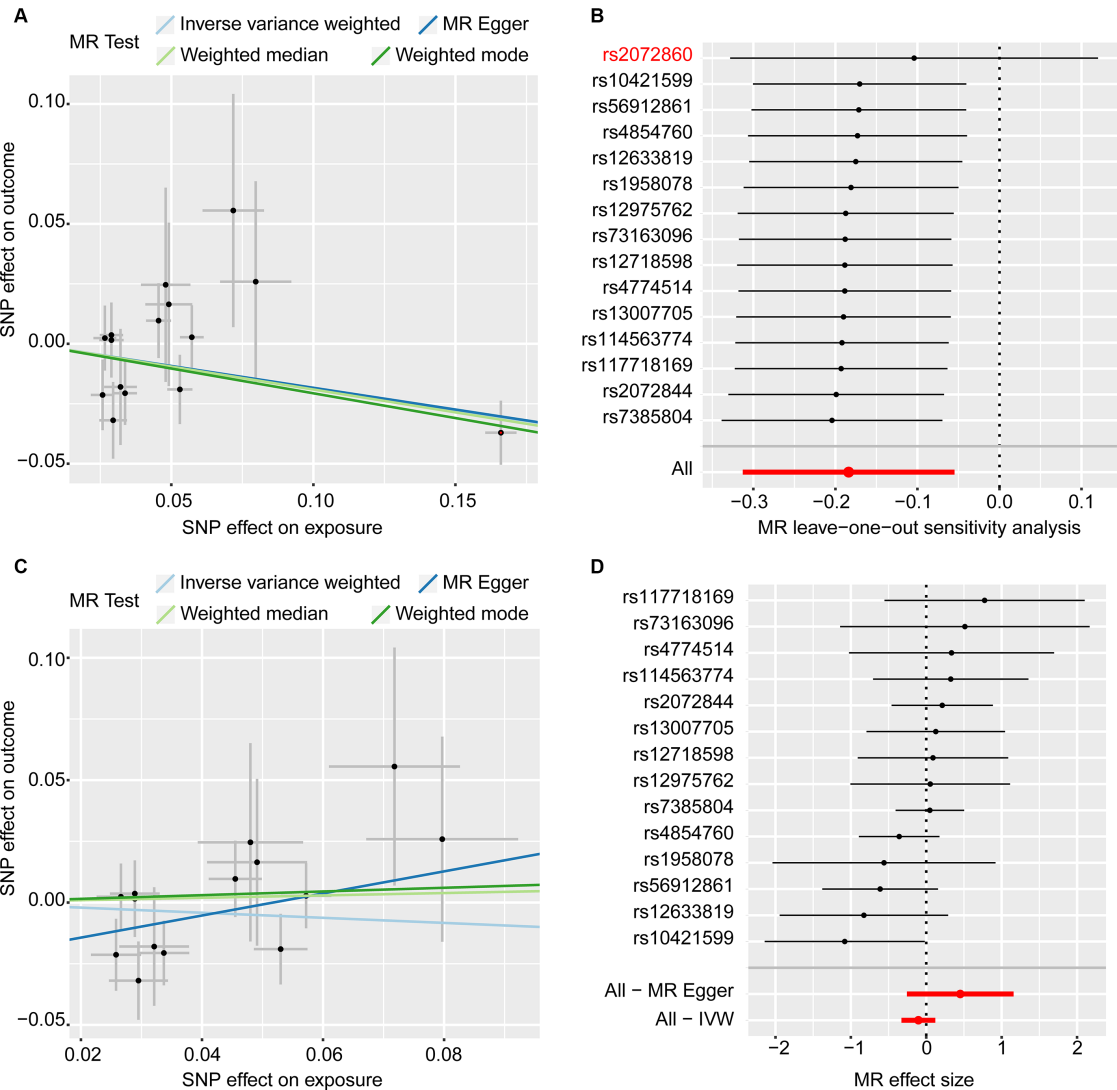
MR analyses of the causal effects of serum iron on female infertility. **(A)** Scatter plots (15 SNPs). **(B)** Leave-one-out plot (15 SNPs). **(C)** Scatter plots (14 SNPs). **(D)** Forest plot (14 SNPs).

**Figure 3:**
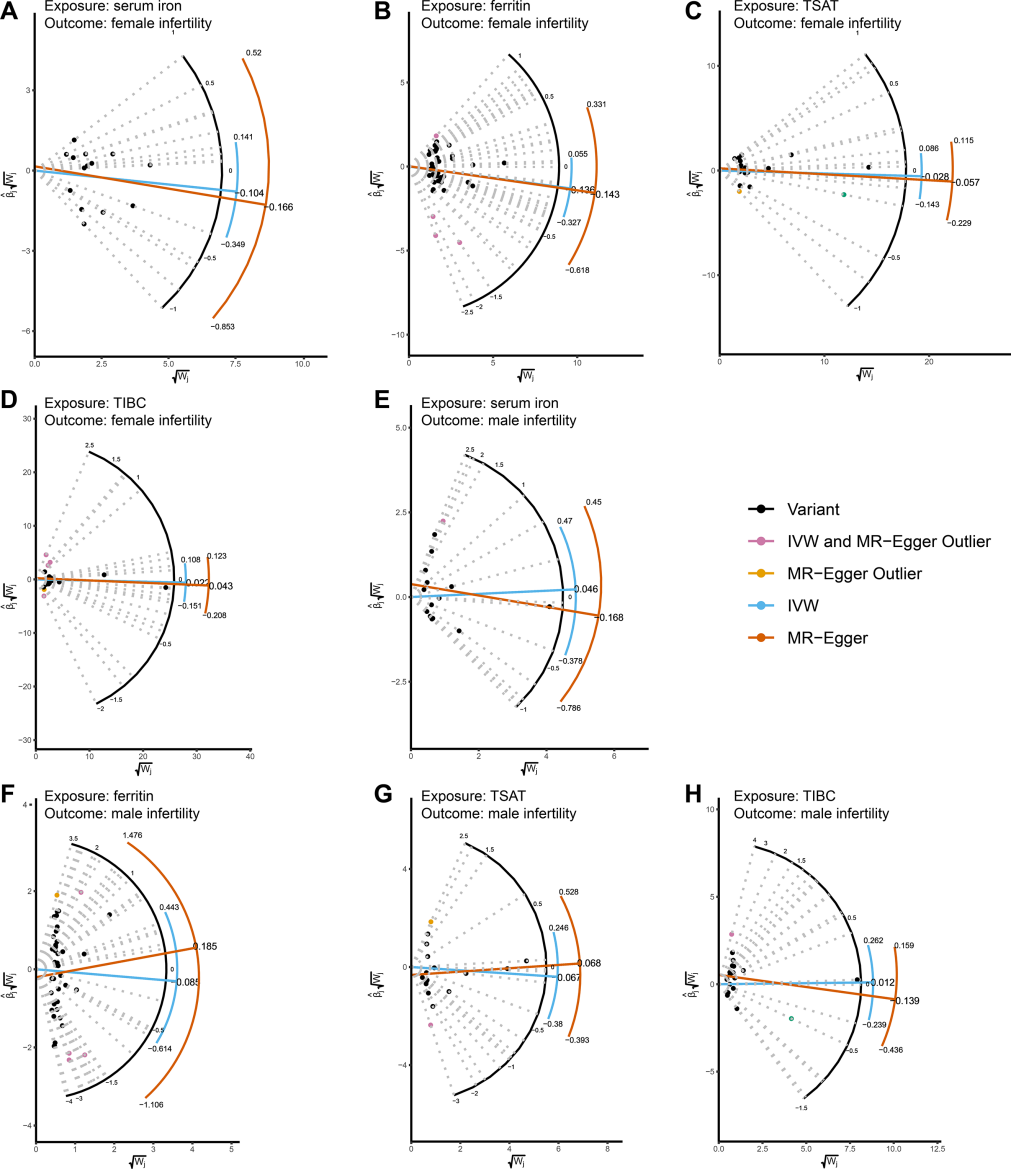
Radial plots showing MR estimates of iron status and infertility. **(A-D)** MR radial plots with female infertility as an outcome and serum iron, ferritin, TSAT, and TIBC as exposures. **(E-H)** Using male infertility as an outcome, serum iron, ferritin, TSAT, and TIBC were used as exposed MR radial plots. Both IVW and radial MR-Egger regressions were conducted using the RadialMR software package. The horizontal axis $\left({\sqrt{W}}_{j}\right)$ of the Radial plot is the square root of the actual weight obtained for each SNP in the MR analysis, while the vertical axis $\left(\wedge \beta_{j}\;{\sqrt{W}}_{j}\right)$ represents the ratio estimates for each SNP multiplied by the same square root weight. Outliers (depicted in yellow and pink) are identified for both methods, while variants (valid SNPs) are represented by black dots. The curves display ratio estimates for each SNP (blue for IVW methods, orange for MR-Egger methods). TSAT, transferrin saturation; TIBC, total iron binding capacity.

The results of the MR analysis that investigated the causal link between iron status and infertility were shown in Table 1. However, our analysis found no significant evidence of genetic causality between iron and infertility. In our analysis of the causal association between serum iron with female infertility, we rigorously screened the instrumental variables according to the three conditions described in the Methods section, and subsequently, 15 SNPs were used for MR analysis. Our analysis revealed that serum iron tended to show a negative causal association with female infertility; however, although the MR-Egger analysis did not reach statistical significance (*p* = 0.116, se = 0.108), IVW (*p* = 0.005, se = 0.066), weighted median (*p* = 0.011, se = 0.075), and weighted mode (*p* = 0.019, se = 0.078) analyses consistently indicated a negative causal association between serum iron and female infertility (Table 1; Figure 2A). However, it seems that this negative causal association was mainly influenced by one SNP, rs2072860, which is located in the known iron-associated gene TMPRSS6, as indicated by the scatterplot and leave-one-out plots (Figures 2A,B). To avoid the influence of a single SNP on our MR results, we excluded this SNP and re-ran the MR analysis using the remaining 14 SNPs as instrumental variables. Ultimately, our analysis showed no causal association between serum iron levels and female infertility (Table 1; Figures 2C,D). In addition, ferritin (*p*-IVW = 0.151, se = 0.095), TSAT (*p*-IVW = 0.600, se = 0.054), and TIBC (*p*-IVW = 0.727, se = 0.062) did not indicate a significant causal association with female infertility (Table 1). Similar to the findings regarding female infertility, our analysis did not observe a meaningful causal association between iron status and male infertility (Table 1). Additionally, radial plots for IVW and MR-Egger analysis were generated, and several outliers were identified (Figure 3). Nevertheless, the results still did not reach statistical significance after removing the outliers (results not shown).

**Table 1:** MR studies of the causal link between body iron status and infertility.

Exposure	n SNP	Method	Outcome: female infertility			Outcome: male infertility		
			b	se	*p*-val	b	se	*p*-val
Serum iron	15	IVW	−0.184	0.066	0.005	0.046	0.200	0.817
	15	MR Egger	−0.181	0.108	0.116	−0.253	0.321	0.444
	15	Weighted median	−0.191	0.075	0.011	−0.069	0.232	0.768
	15	Weighted mode	−0.206	0.078	0.019	−0.115	0.235	0.632
	14	IVW	−0.104	0.114	0.363	/	/	/
	14	MR Egger	0.451	0.361	0.235	/	/	/
	14	Weighted median	0.048	0.170	0.776	/	/	/
	14	Weighted mode	0.075	0.208	0.722	/	/	/
Ferritin	43	IVW	−0.136	0.095	0.151	−0.085	0.262	0.745
	43	MR Egger	−0.101	0.180	0.577	0.260	0.491	0.599
	43	Weighted median	0.022	0.120	0.856	0.321	0.354	0.365
	43	Weighted mode	−0.056	0.135	0.681	0.600	0.491	0.229
TSAT	17	IVW	−0.028	0.054	0.600	−0.067	0.147	0.650
	17	MR Egger	−0.012	0.081	0.880	0.058	0.216	0.793
	17	Weighted median	−0.007	0.066	0.916	0.006	0.166	0.972
	17	Weighted mode	−0.016	0.058	0.788	−0.001	0.151	0.994
TIBC	20	IVW	−0.022	0.062	0.727	0.012	0.120	0.921
	20	MR Egger	−0.052	0.079	0.521	−0.203	0.132	0.142
	20	Weighted median	−0.039	0.040	0.327	0.025	0.126	0.842
	20	Weighted mode	−0.044	0.037	0.255	−0.030	0.104	0.780

TSAT, transferrin saturation; TIBC, total iron binding capacity; IVW, Inverse variance weighted.

### Sensitivity analysis

To validate the reliability of the IVW results, sensitivity analyses were conducted and presented in Table 2. In the analysis of the causal association between serum iron and infertility, there was neither heterogeneity (*p* > 0.05) nor horizontal pleiotropy (*p* > 0.05) detected in the MR analysis. Similarly, in analyzing the association between TSAT and infertility, no heterogeneity or pleiotropy was detected. These findings imply that the results of the above MR analysis are reliable. However, heterogeneity was observed when analyzing the causal association of ferritin with infertility (*p* < 0.05), and although the MR-Egger regression analysis did not show pleiotropy (*p* > 0.05) the MR-PRESSO analysis suggested that there might be potential pleiotropy (*p* < 0.05). Whereas similar results existed when analyzing the association of TIBC with infertility. These findings suggest that the results of the latter two sets of analyses are not very robust (Table 2). It is worth noting out that some outliers were detected while performing the MR-PRESSO analysis, and after removing them and rerunning the MR analysis, significant results were still not obtained (Supplementary Table S2).

**Table 2:** Sensitivity analysis of the MR studies.

Exposure	Outcome	*p*-heterogeneity	*p*-pleiotropy	*p*-MR-PRESSO
Serum iron	female infertility	0.462	0.130	0.482
	male infertility	0.479	0.253	0.522
Ferritin	female infertility	0.003	0.820	0.003
	male infertility	0.045	0.410	0.037
TSAT	female infertility	0.142	0.789	0.173
	male infertility	0.335	0.438	0.423
TIBC	female infertility	2.00E-06	0.538	0.018
	male infertility	0.171	0.015	0.277

TSAT, transferrin saturation; TIBC, total iron binding capacity; *p*-heterogeneity, which calculated by inverse variance weighted method. *p*-pleiotropy, which calculated by MR-Egger regression tests.

## Discussion

In this MR investigation, we aimed to identify the potential causal link between systemic iron status and infertility. This study is, to our knowledge, the first attempt to explore this association using a genetic epidemiological approach. Our findings indicate that there is no significant genetic causal relationship between four iron status indicators (serum iron, ferritin, TSAT, and TIBC) and both male and female infertility.

Our conclusions markedly differ from those of previous biological studies. In fact, an increasing number of researchers have focused on the relationship between iron status and female infertility. Iron deficiency poses a significant global health challenge, particularly for women (41). A recent observational study showed that iron status is associated with unexplained infertility in women, especially when serum ferritin levels are below 30 μg/L (considered iron deficient) (13). A comprehensive prospective study spanning 8 years and involving 18,555 premenopausal women showed a significant reduction in the likelihood of infertility in females who consumed iron supplements (42). Another animal experiment produced comparable results, revealing significantly lower pregnancy rates in rats with iron deficiency consuming low-iron foods (43). Potential mechanisms underlying female infertility due to iron deficiency may involve impaired follicular development or ovulation (44), abnormal endometrial function (45), and impaired immune function (46). Additionally, iron is a determinant of healthy fetal delivery. The high iron requirements of the metabolically active placenta (47, 48) and the rapidly growing fetus (49) demonstrate the important role of iron in successful pregnancy and fetal development. While the above studies highlight the impact of iron deficiency on female infertility, it is important to note that iron overload can also have adverse effects on female pregnancy. A recent review provided a thorough overview of the correlation between iron overload and various female reproductive health issues, including hypogonadism, toxicity to preimplantation embryos, reduced endometrial tolerance, and infertility-related disorders such as polycystic ovary syndrome and endometriosis (17). The mechanisms underlying female infertility due to iron overload may involve oxidative stress, ovarian dysfunction, and endocrine disruption (17, 50). In summary, there is a broad and complex association between iron status and female infertility in biological studies. Our findings contrast with these observations, potentially due to the nonlinear nature of the causal link between iron markers and infertility. This is evidenced by the U-shaped associations identified in prior observational studies (51).

Our results did not indicate a causal association between iron status and male infertility. Despite this, the existing literature highlights the essential role of iron in male reproductive function (52). Iron is crucial for maintaining ejaculate motility and sperm pH within a functional range (53). Imbalances in iron levels, whether deficient or excessive, have been linked to negative effects on semen quality. Iron, as a component of antioxidant enzymes, guards against reproductive disorders by mitigating oxidative stress (54–56). Conversely, excess iron may contribute to compromised semen quality through heightened levels of reactive oxygen species and subsequent lipid peroxidation (57, 58). A recent study also found that serum iron and ferritin were associated with reproductive hormones produced by the anterior pituitary gland in infertile men, and that these hormones may play an important role in processes such as spermatogenesis and testosterone production (59). While the existing literature underscores the significance of iron in male reproductive health, our study focused on diagnosing male infertility using azoospermia or oligozoospermia criteria. This suggests that there is no apparent causal link between iron and male azoospermia or oligozoospermia (24). However, to gain a more nuanced understanding of the relationship between iron status and male reproduction, additional research is warranted.

The study’s strengths lie in the effective use of MR, a robust method for assessing causal associations while minimizing confounding factors. Additionally, the utilization of publicly available genome-wide association study data with a large sample size enhances the identification of reliable genetic variations and, consequently, the generation of more robust causal associations. Notably, this research represents the first attempt to employ MR in unveiling a causal association between iron status and infertility. However, limitations are acknowledged. Although MR methods control most of the known confounders, there may still be some unknown hidden confounders affecting the results. Heterogeneity of IVs in MR analyses for specific iron status indicators and outcomes was observed, although this was addressed through random effects IVW analyses. The presence of pleiotropy in some analyses, with inconsistent results from MR-PRESSO and MR-Egger regression, underscores the need for cautious interpretation, given the different assumptions and models employed by these methods to handle pleiotropy. The results derived from diverse methods should be synthesized, and efforts to identify more suitable IVs should be pursued in future studies to mitigate the impact of heterogeneity and pleiotropy on MR outcomes. In addition, considering that both iron deficiency and iron excess are detrimental to health, subsequent studies need to focus on the nonlinear causal association of iron markers with infertility. Moreover, the applicability of our results to non-European populations requires additional investigation. Genetic variations and dietary disparities across races and geographical regions may influence individual iron status, consequently impacting infertility outcomes.

## Conclusion

In summary, our findings indicate that, from a genetic perspective, there is no evident causal link between the four indicators of iron homeostasis and infertility. However, the critical role of iron in reproductive health should not be dismissed, and the two may have unknown associations at other levels. The complex etiology of infertility and the potential pleiotropy of our genetic instrumentation need to be interpreted with caution. For future investigations, we propose utilizing larger and more diverse cohorts, along with refined instrumental variables to better address confounding factors.

## Data availability statement

The original contributions presented in the study are included in the article/Supplementary material, further inquiries can be directed to the corresponding author.

## Author contributions

LG: Conceptualization, Data curation, Formal analysis, Investigation, Methodology, Software, Validation, Visualization, Writing – original draft, Writing – review & editing. SY: Data curation, Writing – original draft. HW: Writing – review & editing, Resources, Supervision. JP: Writing – review & editing, Funding acquisition, Project administration, Supervision.

## Footnotes

^1^ https://r9.finngen.fi/

^2^ http://www.phenoscanner.medschl.cam.ac.uk
