# Supplementary material for: No evidence of genetic causation between iron and infertility: a Mendelian randomization study
Source: Front Nutr. 2024 Jul 22;11:1390618. doi: 10.3389/fnut.2024.1390618 (PMC11298439; doi:10.3389/fnut.2024.1390618)
Supplement: Supplementary file 1 [file Table_1.docx]

Supplementary Table S1. Basic information on exposure and outcome data.

| Trait | | Cohort(s) or Consortium | Source | Sample size or Cases/Control |
| --- | --- | --- | --- | --- |
| Exposures | serum iron | Iceland, the UK, and Denmark | Bell et al., 2021 | 163,511 |
|  | ferritin |  |  | 246,139 |
|  | TSAT |  |  | 131,471 |
|  | TIBC |  |  | 135,430 |
| Outcome | female infertility | FinnGen | https://r9.finngen.fi/ | 13,142/107,564 |
|  | male infertility |  |  | 1271/119,297 |
