# Supplementary material for: No evidence of genetic causation between iron and infertility: a Mendelian randomization study
Source: Front Nutr. 2024 Jul 22;11:1390618. doi: 10.3389/fnut.2024.1390618 (PMC11298439; doi:10.3389/fnut.2024.1390618)
Supplement: Supplementary file 2 [file Table_2.docx]

Supplementary Table S2. Selection and filtering of instrumental variables.

| Parameters | | Serum iron | Ferritin | TSAT | TIBC |
| --- | --- | --- | --- | --- | --- |
| SNP filter | Number of significant SNP | 8777 | 7813 | 13944 | 20193 |
|  | Number independent SNP | 16 | 51 | 19 | 26 |
|  | Remove confounding SNPs | 15 | 43 | 17 | 20 |
| Outcome: female infertility | *P*-MR-PRESSO | 0.556 | 0.003 | 0.173 | 0.018 |
|  | *P*-IVW | 0.005 | 0.151 | 0.600 | 0.727 |
|  | Outliers SNPs | 1 | 2 | / | 4 |
|  | Remove outliers SNPs *P*-IVW | 0.363 | 0.671 | / | 0.747 |
| Outcome: male infertility | *P*-MR-PRESSO | 0.522 | 0.037 | 0.423 | 0.277 |
|  | *P*-IVW | 0.817 | 0.745 | 0.65 | 0.921 |
|  | Outliers SNPs | / | / | / | / |
|  | Remove outliers SNPs *P*-IVW | / | / | / | / |
